# Development of a GC-MS Method for Determination of Various Polycyclic Aromatic Hydrocarbons in Iranian Traditional and Semi-industrial Taftoon Bread

**DOI:** 10.22037/ijpr.2019.15323.13018

**Published:** 2020

**Authors:** Vahideh Moradi, Seyed Mahdi Seyedain Ardabili, Attaollah Shakoori, Seyed Ebrahim Hoseyni

**Affiliations:** a *Department of Food Science and Technology, Science and Research Branch, Islamic Azad University, Tehran, Iran. *; b *Vice-Chancellor for Food and Drug Affairs Shahid Beheshti University of Medical Sciences, Tehran, Iran. *; c *Food Safety Research Center, Shahid Beheshti University of Medical Sciences, Tehran, Iran.*

**Keywords:** GC-MS, Polycyclic Aromatic Hydrocarbons (PAHs), Taftoon bread, Iran

## Abstract

This research reports a validated multi-residue method based on gas chromatography coupled mass spectrometry technique for analysis of 24 Polycyclic Aromatic Hydrocarbons (PAHs) in traditional and semi-industrial Taftoon bread using QuEChERS sample preparation.

Matrix effect studies were performed by comparing the slopes of solvent based standard calibration curves and spiked calibration curves. Due to enhancement or suppression effects of matrix, validation of the method was performed using spiked calibration curves. In the concentration range of 10-500 ng/g, the calibration curves for each analyte was linear with a determination coefficient (R^2^) of 0.991-0.999. The Detection and quantitation limits for the studied PAHs were calculated 0.14-1.49 ng/g and 0.46-4.91 ng/g. The average recoveries for three spiked levels (25, 50 and 200 ng/g), were in the range 77-103% (n = 27), with a satisfactory precision (RSD < 20%).

Analysis of Taftoon bread samples using the validated method showed that three compounds; NPH, PHE and ANT were found in 37 (35.2%) samples and in the term of traditional and semi- industrial samples the occurrence of mentioned PAHs were 36.1% and 33.3%, respectively. According to the findings, we proposed that direct flame exposure in gas oven during baking of Taftoon bread could produce PAHs in bread samples.

## Introduction

Polycyclic aromatic hydrocarbons (PAHs) are a large class of highly lipophilic compounds containing fused aromatic ring. Chemically, they can be divided into light (2-4 rings) and heavy (5 or more rings) groups on the basis of the number of condensed aromatic rings. It has been known that the heavy PAHs, such as benzo[a] pyrene, dibenzo[a,h] anthracene, benzo [g,h,i] perylene, and indeno [1,2,3-c,d] pyrene are more stable and more toxic than the light ones (1, 2). Numerous organizations for example the International Agency for Research on Cancer (3), the European Food Safety Authority (4) and the others consider PAHs to be toxic compounds with carcinogenic, teratogenic, and mutagenic properties.

According to the studies on PAHs exposure, food is the main source of human exposure to PAHs. Food contaminated with PAHs generally arises from environmental contamination (including Polluted water and soil, agricultural burning and Post-harvest), food processing, contaminated packaging, and direct contact with non-food grade mineral oil (5-10).

As shown, PAHs can occur in various foods and cereals are one of the major sources (10-12). Wheat, as an important cereal is produced in the worldwide and widely consumed in different forms, including bread. Bread is prepared from wheat-flour dough then cultured with yeast, and finally baked in various methods such as oven. 

Some international investigations have shown that PAHs can be occurred in bread in different ways, both through the environmental pollutants and during baking processes (10-17). Because of daily and high per capita consumption, occurrence of PAHs in bread can cause a major concern for human health. 

Nowadays, various methods have been established for Analysis of PAHs in food matrixes based on chromatographic detections. Numerous extraction methods have been introduced to removal of PAHs in foods, like the lipid extraction methods, followed by various preparation techniques (7). Instrumentally, the most commonly reported technique for analysis of PAHs is gas chromatography (GC) coupled different detectors. GC coupled mass spectrometry (MS) detector owing to high selectivity and sensitivity is a powerful instrument for detection and trace analysis of PAHs in foods and other commodities. Molecularly, PAHs are very stable compounds, and in the ionization process, they are mainly fragmented to the molecular ion ([M-H]‏ or [M-2H]). Thus, the single MS (quadrupole) are appropriate technique for analysis of PAHs in food samples (7, 12 and 18). Few studies reported the use of GC- tandem mass spectrometry (GC-MS/MS) or GC- high-resolution mass spectroscopy (GC-HRMS) for analysis of PAHs in various matrixes (19, 20). 

Bread is one of the most valuable foodstuffs around the world (21). It is the main food of the Iranian people, and cooked by using traditional and industrial methods. Traditional Iranian breads are very popular and renowned for their taste. Because of ethnic and racial variety, many types of traditional breads are baked in different regions of Iran such as, Taftoon, Lavash, Barbari, and Sangak. Taftoon is categorized as flat circle bread and produced by mixing wheat flour, water, salt, and leaven. Previously, Taftoon was baked in a clay oven, but in recent decades it is mainly prepared in direct gas fired machines (22). 

 In Iran, a few investigations have been conducted on occurrence of PAHs in breads especially traditional breads. Recently, Eslamizad *et al.* developed a modified QuEChERS extraction for detection and determination of benzo[a]pyrene (BaP), the well-known member of PAHs, in traditional, semi-industrial, and industrial Sangak bread samples (23). They detected different amounts of BaP in the collected samples. In this study, only BaP was investigated and other PAHs were not conducted. Due to great consumption of Taftoon in Iran, development a new mothed for assessment of BaP and other PAHs in Taftoon bread samples is required.

In this investigation, a reliable method was developed for analysis of 24 PAHs in traditional Taftoon bread using GC-MS then, the established method was used to detection of studied PAHs in Taftoon bread samples collected in various different of Tehran.

## Experimental

*Chemicals and reagents*

A 15 mixed standards of PAHs including; benzo[c]fluorene (B[c]F,97%), benzo[a]anthracene (B[a]A,99%), chrysene (CHR, 99%), 5-metylchrysene (5-MChr,99%), cyclopenta[c,d]pyrene (C[cd]P,99%), benzo[b]fluoranthene (B[b]F,99%), benzo[k]fluoranthene (B[k]F,99%), benzo[a]pyrene (B[a]P,99%), dibenzo[a,h]anthracene (D[ah]A, 99%), indeno[1,2,3-cd]pyrene (I[cd]P, 99%), benzo[g,h,i]perylene (B[ghi]P, 99%), dibenzo[a,l]pyrene (D[al]P, 99%), dibenzo[a,e]pyrene (D[ae]P, 99%), dibenzo[a,i]pyrene (D[ai]P, 99%) and dibenzo[a,h]pyrene (D[ah]P, 99%) were provided from Restek company (United States) and 9 single chemicals including; Phenanthrene (PHE, 99.20%), Naphthalene (NPH, 99.40%), Fluorene (FLR, 99.90%), Fluoranthene (FLA, 98.7%), Pyrene (PYR, 98%), Anthracene (ANT, 99.40%), Acenaphthene (ACP, 99.9%), Perylene (PER, 99.9%), Acenaphtylene (ACL, 99.9%) were prepared from Sigma Aldrich (United states). Stock standards of 9 single PAHs were prepared at concentration 1000.0 μg/mL in Ethyl acetate then; a mixed solution of all PAHs standards was made ready by dissolving appropriate concentration of 15 mixed standards and 9 single standards in acetonitrile at a concentration of 5.0 μg/mL. Primary and working standards were prepared from the final mixed standard. All standard solutions were stored in amber flasks at -20 °C.

Triphenylphosphate (TPP), as internal standard was obtained from Sigma–Aldrich (Germany). A stock solution of TPP in ethyl acetate at concentration of 10.0 μg/mL was prepared and a 50.0 μL of its solutions was added to the spiked bread samples. Anhydrous magnesium sulfate (MgSO_4_) was acquired from Sigma–Aldrich (Germany) and Methanol (MeOH) and HPLC-grade acetonitrile (MeCN) from Acros (Belgium). Ethyl acetate (EtAc) and sodium acetate were supplied from Merck (Darmstadt, Germany). Bondesil-primary secondary amine (PSA, 40 μm) was provided from Interchim (France). A Milli-Q Plus ultra-pure water system (Molsheim, France) was applied for preparation of HPLC grade water. 

*Instrument Analysis*

All samples were determined using an Agilent 7890A GC instrument coupled a 5975C mass detector with split/spiltless injector, and 7693 autosampler (Agilent technologies, USA). A DB-5MS 122-5532UI capillary column (30 m × 0.25 mm I.D., 0.25-μm film) from Agilent technologies and Helium carrier gas (purity 99.999%) at a constant flow rate of 1.6 mL/min with the following oven temperature program was applied: 80 °C (2 min), 20 °C/min ramp to 140 °C (1 min), then 5 °C/min ramp to 315 °C. Injection port, quadrupole mass analyser, transfer line and ion source was adjusted at 300 °C, 100 °C, 280 °C, and 230 °C, respectively and splitless mode was used. A mass range of m/z 50-500 was scanned to find the retention time and diagnostic ions (quantification and confirmation ions) of the analytes. After acquisition of the diagnostic ions in selected ion monitoring (SIM) mode, the retention times and mass spectra of selected ions were used to identification of peaks. At least three ions were used to recognition and determination of analytes. The most abundant ions with the highest signal-to-noise ratios were chosen as quantifiers and the others were qualifiers. All identified peaks were confirmed by comparing the relative abundances of studied ions of PAHs to the related spectra in the mass reference library. 

*Sample preparation*

Sample preparation including, extraction and clean-up was performed according to the QuEChERS method (24). The bread samples were carefully ground and homogenized, then a 5 g portion of the obtained powder was transferred into a 50 mL falcon tube. The desired amounts of the mixed PAH standard (for spiking) and 50 µL TPP (200 ng/mL) were included to the tube. Thereafter, by adding 14 mL of MeCN, the studied PAHs were taken out bread. The tube content was vortex mixed for 3.0 min, then 2 g anhydrous MgSO_4_ and 1.5 g sodium acetate were added and after mixing for another 3.0 min the content was introduced to a centrifuge 9055×g for 20 min. After centrifugation, the 7 mL of supernatant was taken into a suitable tube and evaporated until dryness by a nitrogen evaporator. The remainder was reconstituted in 0.5 mL MeCN and sonicated 10.0 min then vortex mixed for 3.0 min. The obtained solution was transferred to a micro tube containing 60 mg anhydrous MgSO4 and 20 mg PSA and vortex mixed vigorously for 1min and centrifuged for 5 min 18894×g. Finally, a desired amount of aliquot was transferred into a screw cap vial and 2.0 μL was injected into GC-MS.

*Method validation*

For validation studies, various parameters including; linearity, recovery, precision, limits of detection (LOD) and limits of quantification (LOQ) were calculated (25-27). Linearity was assessed applying spiked calibrations by analyzing in triplicate six concentration levels, between 10 and 500 ng/g. To estimation of the accuracy (recovery studies) and the precision, three spiked blank bread samples at concentration levels of 25, 50, and 200 ng/g were prepared. The matrix effects were calculated as: [1 - (spiked calibration curve slope/solvent base calibration curve slope)] ×100 (24). LODs and LOQs were estimated according to the concentrations of PAH resulting in a signal-to-noise ratio of 3 and 10, respectively. The amounts of PAHs in Taftoon samples were calculated by interpolation of the peak areas for each PAH to internal standard peak area in the sample.

*Determination of PAHs in Taftoon samples*

Seventy-two traditional and thirty-three semi- industrial Taftoon bread samples collected from Taftoon bakeries located in Tehran city. Traditional bread were baked by direct heating in the temperature range of 216-300 °C and semi-industrial bread were baked by indirect heating at temperatures between 160 and 300 °C in different distance of heating source. Natural gas had been used for both of the oven. After collection, all of the samples were covered with aluminum foil and transported to the lab. Each sample was coded and dried to lose its moisture within one day. Then all of the samples were ground and stored in amber glass bottles at *−*20 °C until analysis. Finally, 50 g portion of homogenized samples was weighted and analyzed. 

**Table 1:** Molecular weights, quantification and confirmation ions, ion ratio and retention times of studied PAHs

**NO.**	**Compounds**	**Molecular** **Weights (g/mol)**	**Quantification Ions (m/z)**	**Confirmation Ions (m/z)**	**Ion Ratios**	**Retention Times (min)**
1	NPH	128	128	128 ,129^*^,64	8.393	5.19
2	ACL	152	152	152,151^*^,76	4.509	8.18
3	ACP	154	153	153,154^*^,76	3.231	8.63
4	FLR	166	166	166,165^*^,82	1.176	10.16
5	PHE	178	178	178,176^*^,152	5.13	12.91
6	ANT	178	178	178,176^*^,152	8.441	13.09
7	FLA	202	202	202, 203^*^,201	6.76	17.40
8	PYR	202	202	202, 203^*^,201	6.546	18.27
9	B(c)F	216	216	216, 215^*^,217	1.349	20.17
10	CP(c,d)P	226	226	226, 227^*^,225	8.634	23.49
11	B(a)A	228	228	228.226^*^,229	2.906	23.66
12	CHR	228	228	228.226^*^,229	3.456	23.79
13	5-M-CHR	242	242	242, 241^*^,239	2.158	25.82
14	B(b)F	252	252	252, 253^*^,250	5.106	28.25
15	B(k)F	252	252	252, 253^*^,250	3.431	28.35
16	B(a)P	252	252	252, 253^*^,250	6.624	29.76
17	PER	252	252	252, 253^*^,250	4.613	29.96
18	I(1,2,3-cd)P	276	276	276, 277^*^,138	5.071	33.45
19	DB(a,h)A	278	278	278, 276^*^,138	3.733	33.67
20	B(g,h,i)PER	276	276	276, 138^*^,277	20.08	34.25
21	DB(a,l)P	302	302	302, 303^*^,300	4.329	37.84
22	DB(a,e)P	302	302	302, 303^*^,300	4.784	38.84
23	DB(a,i)P	302	302	302, 303^*^,300	4.395	39.19
24	DB(a,h)P	302	302	302, 303^*^,300	3.151	39.37

^*^Selected confirmation ion.

**Table 2:** Matrix effects of studied PAHs in spiked and solvent-based calibration curves

**NO.**	**Compound**	**Spiked calibration curve of bread**		**Solvent ** **calibration** **curve**		**A**	**Matrix Effect (%)**
		**Slope**	**R** ^2^	**Slope**	**R** ^2^		
1	NPH	0.0002	0.989	0.009	0.998	0.022	97.78
2	ACL	0.002	0.998	0.023	0.997	0.098	90.15
3	ACP	0.002	0.998	0.026	0.991	0.088	91.19
4	FLR	0.005	0.997	0.020	0.999	0.249	75.12
5	PHE	0.005	0.998	0.010	0.994	0.490	50.94
6	ANT	0.003	0.998	0.008	0.999	0.410	58.98
7	FLA	0.007	0.999	0.018	0.994	0.379	62.13
8	PYR	0.007	0.996	0.041	0.992	0.175	82.47
9	B(c)F	0.006	0.999	0.059	0.999	0.103	89.72
10	CP(c,d)P	0.006	0.996	0.015	0.993	0.375	62.48
11	B(a)A	0.005	0.999	0.004	0.999	1.366	-36.64
12	CHR	0.006	0.999	0.013	0.998	0.423	57.65
13	5-M-CHR	0.005	0.999	0.014	0.995	0.352	64.80
14	B(b)F	0.008	0.999	0.012	0.999	0.705	29.52
15	B(k)F	0.003	0.999	0.009	0.999	0.341	65.91
16	B(a)P	0.006	0.992	0.016	0.995	0.393	60.71
17	PER	0.007	0.999	0.026	0.997	0.265	73.48
18	I(1,2,3-cd)P	0.006	0.998	0.014	0.993	0.422	57.79
19	DB(a,h)A	0.005	0.999	0.002	0.990	2.174	-117.38
20	B(g,h,i)PER	0.009	0.998	0.015	0.999	0.616	38.44
21	DB(a,l)P	0.004	0.998	0.014	0.997	0.271	72.93
22	DB(a,e)P	0.004	0.999	0.025	0.994	0.157	84.27
23	DB(a,i)P	0.003	0.996	0.025	0.994	0.127	87.33
24	DB(a,h)P	0.002	0.998	0.026	0.994	0.078	92.15

**Table 3:** Regression equations and coefficients of determination (R^2^), LOQs, LODs (ng/g) obtained for studied PAH in bread samples

**NO.**	**Compound**	**Regression Equation (n = 18)**	**Coefficients of Determination (R** ^2^ **)**	**LOD** ^a^	**LOQ** ^b^
1	NPH	y = 0.0003x + 0.009	0.99	1.49	4.91
2	ACL	y = 0.002x + 0.038	0.998	0.65	2.50
3	ACP	y = 0.025x + 0.134	0.991	0.65	2.15
4	FLR	y = 0.005x + 0.052	0.997	0.75	2.48
5	PHE	y = 0.005x + 0.077	0.998	0.61	2.01
6	ANT	y = 0.003x + 0.011	0.998	0.56	1.85
7	FLA	y = 0.006x - 0.006	0.999	0.14	0.46
8	PYR	y = 0.007x - 0.037	0.996	0.87	2.88
9	B(c)F	y = 0.006x - 0.002	0.999	0.18	0.60
10	CP(c,d)P	y = 0.005x - 0.002	0.996	0.82	2.71
11	B(a)A	y = 0.005x + 0.012	0.999	0.24	0.80
12	CHR	y = 0.013x - 0.068	0.998	0.20	0.67
13	5-M-CHR	y = 0.005x + 7E-05	0.999	0.24	0.80
14	B(b)F	y = 0.008x + 0.015	0.999	0.35	1.15
15	B(k)F	y = 0.003x + 0.008	0.999	0.39	1.28
16	B(a)P	y = 0.017x - 0.106	0.994	0.79	2.59
17	PER	y = 0.006x + 0.012	0.999	0.39	1.29
18	I(1,2,3-cd)P	y = 0.006x + 0.021	0.998	0.56	1.85
19	DB(a,h)A	y = 0.005x + 0.004	0.999	0.36	1.19
20	B(g,h,i)PER	y = 0.009x + 0.072	0.998	0.58	1.90
21	DB(a,l)P	y = 0.014x - 0.057	0.997	0.59	1.96
22	DB(a,e)P	y = 0.004x + 0.011	0.999	0.30	1.52
23	DB(a,i)P	y = 0.003x + 0.020	0.996	0.84	2.76
24	DB(a,h)P	y = 0.002x + 0.027	0.998	0.57	1.88

^a^LOD: limit of detection for a S/N = 3.

^b^LOQ: limit of quantification for a S/N = 10.

**Table 4:** Recoveries (%) and relative standard deviations (RSD, %) obtained for studied PAHs in Taftoon bread samples, spiked at 25, 50 and 200 ng/g levels (n = 9).

**NO.**	**Compound**	**25 (ng/g)**		**50 (ng/g)**		**200 (ng/g)**		**Average recovery** **(n = 27)**	**Average** **RSD** **(n = 27)**
		**Recovery**	**RSD**	**Recovery**	**RSD**	**Recovery**	**RSD**		
1	NPH	117.72	17.28	111.36	10.83	71.14	18.69	100.07	15.60
2	ACL	98.21	4.77	111.72	6.89	99.18	10.81	103.04	7.49
3	ACP	114.96	6.58	108.04	9.59	75.99	18.35	99.66	11.51
4	FLR	90.37	3.36	92.15	6.14	104.91	5.36	103.04	7.49
5	PHE	89.19	1.39	96.83	6.04	79.89	15.22	88.64	7.55
6	ANT	93.96	1.37	98.26	5.09	80.65	13.47	90.96	6.64
7	FLA	108.52	1.11	107.89	2.31	77.28	10.25	97.89	4.56
8	PYR	115.40	0.94	112.79	1.66	78.38	7.97	102.19	3.52
9	B(c)F	104.73	1.09	105.96	1.76	74.29	9.33	94.99	4.06
10	CP(c,d)P	92.13	1.76	85.25	2.16	64.17	7.38	80.52	3.77
11	B(a)A	86.75	3.58	94.43	2.01	81.26	20.11	87.48	8.57
12	CHR	100.15	1.67	99.95	1.89	72.09	10.20	90.73	4.59
13	5-M-CHR	100.69	1.62	103.70	1.96	73.47	10.67	92.62	4.75
14	B(b)F	93.35	1.93	104.36	2.74	73.24	9.18	90.31	4.62
15	B(k)F	90.31	2.44	98.06	2.56	74.08	13.40	87.49	6.13
16	B(a)P	90.28	3.09	94.18	4.40	67.10	8.70	83.85	5.40
17	PER	96.94	1.99	103.72	2.27	76.38	7.76	92.35	4.01
18	I(1,2,3-cd)P	82.10	1.63	88.71	5.90	61.90	14.53	77.57	7.35
19	DB(a,h)A	116.87	4.42	108.82	5.16	80.93	13.58	102.20	7.72
20	B(g,h,i)PER	91.46	1.71	101.39	3.92	69.57	7.33	87.48	4.32
21	DB(a,l)P	81.52	2.89	100.76	4.83	71.23	6.67	84.51	4.80
22	DB(a,e)P	101.24	4.07	116.09	4.32	73.38	8.36	96.90	5.58
23	DB(a,i)P	75.87	2.95	94.87	5.91	64.38	14.12	78.38	7.66
24	DB(a,h)P	101.42	6.42	108.69	7.43	75.86	37.94	95.33	17.26

**Table 5:** PAHs values determined in traditional Taftoon bread samples (n = 72).

**NO.**	**Compound**	**Numbers of positive samples**	**LOD** **(ng/g)**	**LOQ** **(ng/g)**	**Mean** **(ng/g)**	**Min Level** **(ng/g)**	**Max Level** **(ng/g)**
1	NPH	20 (27.8%)	1.49	4.91	88.68	7.17	201.10
2	ACL	0	0.65	2.50	0	nd	nd
3	ACP	0	0.65	2.15	0	nd	nd
4	FLR	0	0.75	2.48	0	nd	nd
5	PHE	1 (1.4%)	0.61	2.01	2.29	-	2.29
6	ANT	5 (6.9%)	0.56	1.85	12.33	9.82	18.09
7	FLA	0	0.14	0.46	0	nd	nd
8	PYR	0	0.87	2.88	0	nd	nd
9	B(c)F	0	0.18	0.60	0	nd	nd
10	CP(c,d)P	0	0.82	2.71	0	nd	nd
11	B(a)A	0	0.24	0.80	0	nd	nd
12	CHR	0	0.20	0.67	0	nd	nd
13	5-M-CHR	0	0.24	0.80	0	nd	nd
14	B(b)F	0	0.35	1.15	0	nd	nd
15	B(k)F	0	0.39	1.28	0	nd	nd
16	B(a)P	0	0.79	2.59	0	nd	nd
17	PER	0	0.39	1.29	0	nd	nd
18	I(1,2,3-cd)P	0	0.56	1.85	0	nd	nd
19	DB(a,h)A	0	0.36	1.19	0	nd	nd
20	B(g,h,i)PER	0	0.58	1.90	0	nd	nd
21	DB(a,l)P	0	0.59	1.96	0	nd	nd
22	DB(a,e)P	0	0.30	1.52	0	nd	nd
23	DB(a,i)P	0	0.84	2.76	0	nd	nd
24	DB(a,h)P	0	0.57	1.88	0	nd	nd
	∑24 PAHs	26 (36.1%)	-	-	70.67	2.29	201.10

nd: not detected.

**Table 6:** PAHs values determined in semi industrial Taftoon bread samples (n = 33).

**NO.**	**Compound**	**Numbers of positive samples**	**LOD** **(ng/g)**	**LOQ** **(ng/g)**	**Mean** **(ng/g)**	**Min Level** **(ng/g)**	**Max Level** **(ng/g)**
1	NPH	7 (21.2%)	1.49	4.91	46.39	5.09	241.15
2	ACL	0	0.65	2.50	0	nd	nd
3	ACP	0	0.65	2.15	0	nd	nd
4	FLR	0	0.75	2.48	0	nd	nd
5	PHE	1 (3.0%)	0.61	2.01	6.76	-	6.76
6	ANT	3(9.1%)	0.56	1.85	10.89	10.73	11.12
7	FLA	0	0.14	0.46	0	nd	nd
8	PYR	0	0.87	2.88	0	nd	nd
9	B(c)F	0	0.18	0.60	0	nd	nd
10	CP(c,d)P	0	0.82	2.71	0	nd	nd
11	B(a)A	0	0.24	0.80	0	nd	nd
12	CHR	0	0.20	0.67	0	nd	nd
13	5-M-CHR	0	0.24	0.80	0	nd	nd
14	B(b)F	0	0.35	1.15	0	nd	nd
15	B(k)F	0	0.39	1.28	0	nd	nd
16	B(a)P	0	0.79	2.59	0	nd	nd
17	PER	0	0.39	1.29	0	nd	nd
18	I(1,2,3-cd)P	0	0.56	1.85	0	nd	nd
19	DB(a,h)A	0	0.36	1.19	0	nd	nd
20	B(g,h,i)PER	0	0.58	1.90	0	nd	nd
21	DB(a,l)P	0	0.59	1.96	0	nd	nd
22	DB(a,e)P	0	0.30	1.52	0	nd	nd
23	DB(a,i)P	0	0.84	2.76	0	nd	nd
24	DB(a,h)P	0	0.57	1.88	0	nd	nd
	∑24 PAHs	11 (33.3%)	-	-	33.11	5.09	241.15

nd: not detected.

**Figure 1:**
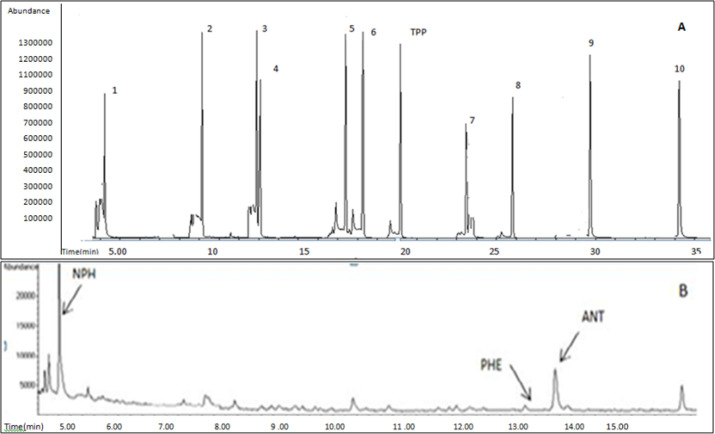
Chromatogram of some PAH standards; (A) TPP as the internal standard, NPH (1); FLR (2); PHE (3); ANT (4); FLA (5); B[c]F (6); 5-M-CHR (7); B[b]F (8); B[g,h,i]PER (9); DB[a,e]P (10). (B) Detected PAHs in aftoon bread samples

## Results and Discussion

*Gas chromatography mass spectrometry determination*

Optimization of GC and MS conditions as a key point in the analysis was accurately conducted. Various GC parameters including oven temperature program, MS conditions, and assess to suitable column were obtained by consecutive injections. Therefore, the SIM mode was applied for analysis of the investigated PAHs. Quantitation and confirmation of PAHs were performed based on the use of: one target as quantification ion, at least two confirmations, their ion ratios and also retention times. Table 1 summarizes molecular weights, retention times, SIM conditions, and calculated ion ratios obtained for the studied PAHs. A chromatogram of some PAH standards (A), and detected PAHs (NPH, PHE and ANT) in Taftoon samples (B) are shown in Figure 1.

*Validation studies*

Validation studies were performed for assessment of linearity, accuracy, precision, and limits of detections (LOD) and quantifications (LOQ). One of the most important challenges in development of an analytical method is matrix effect. In this challenge, components of the sample affect the results of an analysis, altering assay sensitivity and repeatability (28). In food analyses by GC-MS, original components, such as peptides, sugars, and lipids or added ingredients contribute in matrix are effected by interfering gas chromatography and/or mass spectrometry (29). Components of Taftoon bread, like salt, wheat protein, and carbohydrates can lead to suppression or enhancement of targeted ions in mass spectrometry assay. Matrix effect was calculated by comparing the slopes of solvent based calibration curves and spiked calibration curves and declared in terms of ion suppression or enhancement. As shown in Table 2, matrix effect for all of the analytes shows a strong signal enhance (>50%) except matrix effect for BbF and BghiPER considered medium signal enhance whereas that was medium and strong suppression signal in BaA and DBahA, respectively. There are various solutions for overcoming of matrix effects, and in this study spiked calibration standard approach was used. Spiked calibration standards at levels of 10, 25, 50, 100, 200, and 500 ng/g were prepared by the addition of 10, 25, 50, 100, 200, and 500 μL of standard stock solutions with concentration of 5000 ng/mL to 5 g of blank bread samples in each case, respectively. Quantification of the PAH compounds in the bread samples was performed by using an internal standard method. Therefore 50 μL of TPP solution in acetonytril (20,000 ng/mL) was added to the all spiked bread samples. 

Calibration curves showed a linear relationship between the concentration and peak area ratios in the range of 10-500 ng/g with a determination coefficient (R^2^) ranging between 0.991 and 0.999. Therefore, the extraction processes and analytical method had enough efficiency for the determination of PAHs at trace levels. Table 3 shows the values of the validation parameters for analytes quantification of spiked calibration (range 10-500 ng/g of triplicates each, n = 18).

Limits of detection (LODs) and Limits of quantification (LOQs) were calculated based on the signal-to-noise ratio of equal to 3 and 10, respectively. The LODs and LOQs as shown in table 3 were between 0.14-1.49 ng/g and 0.46-4.91 ng/g, respectively.

The mean extraction recoveries were determined by applying the full procedure to triplicate samples in three consecutive days of analysis at three spiking levels including 25, 50, and 200 ng/g with the same operator and laboratory. The percentage of mean recoveries obtained 77-103% for each PAH compound considered as the acceptable range of European Commissions regulation (26). 

Precision was expressed as relative standard deviation (RSD) and calculated like the recovery in different days. The average of relative standard deviations (RSDs) of PAHs in bread were in the range of 3.52-17.26% with a satisfactory precision (RSD<20%) which were in the acceptable range of European Commissions regulation (26). The values of mean recovery and RSD percentage for each spiking level are presented in Table 4.

*Determination of PAHs in Taftoon samples*

The developed method was successfully used for detection and determination of 24 PAHs in various traditional and semi- industrial Taftoon bread samples collected from different region of Tehran, the capital of Iran. Totally, 105 Taftoon samples including, 72 traditional, and 33 semi- industrial were analyzed (Tables 5 and 6). The results showed that three compounds; NPH, PHE, and ANT were found in 37 (35.2%) Taftoon samples and in the term of traditional and semi- industrial samples the occurrence of mentioned PAHs was 36.1% and 33.3%, respectively. As shown in table 5 and 6, NPH was found in 20 (27.8%) traditional Taftoon samples and in 7 (21.2%) semi- industrial samples in the range of 7.17-201.1 and 5.09-241.15 ng/g, respectively. ANT was detected in 5 (6.9%) traditional and in 3(9.1%) semi- industrial samples in the range of 9.82-18.09 and 10.73-11.12 ng/g, respectively. One sample (1.4%) of traditional and 1 sample (3.0%) semi- industrial Taftoon was found to be contaminated with PHE in levels 2.29 ng/g and 6.76, respectively. All obtained results were higher than the legal permissible limits (1.0 ng/g) proposed by the European Union for processed cereal-based foods (30). 

Bread’s contamination by PAHs could be due to both the contamination of bakery raw materials, such as water and primarily flour, and the baking process (23). Previous studies have shown that there are few published papers in field of PAHs in Iranian breads. Al-Rashdan *et al.* studied occurrence of 16 PAHs in different bread samples (15). Seven out of 18 samples were Iranian Bread baked from white flour using gas oven. They found NPH, FLR, and PHE were the most three abundant chemicals found in the studied breads. In addition, they reported that some Iranian bread samples had high levels of NPH and PHE. In Iran, most of traditional breads, including Taftoon and some semi-industrials are baked directly using high flame of gas in a short time. Therefore, we proposed that high levels of NPH and PHE could be because of direct flame gas in oven. These findings are in accordance with Rashdan *et al.* studies. 

Eslamizad *et al*. developed a method for investigating BaP in famous Iranian traditional bread called Sangak (31). The collected and analyzed 29 Sangak bread samples from Tehran’s bread bakeries in 2014. Results showed that two Sangak samples were contaminated with BaP. Their another study showed that 35.5% and 13% of Sangak bread samples collected in Tehran and Shiraz were contaminated with BaP, respectively (23). In current study, BaP was not detected in Taftoon samples. Moreover, it has shown that the presence of PAHs in food is significantly due to heat processes such as smoking, smoke-drying, and grilling. However, environmental pollutants are also considered to be an issue (32). Based on the results of Rey-Salgueiro (13), direct toasting (flame-toasting, coal-grilling or gas oven-toasting) or indirect toasting (electric oven-toasting) would strongly affect in PAH levels in the final product. 

Our results showed that beside the other routes, the detected PAHs in bread samples could be originated from gas oven. The efficiency of this route is dependent on various factors such as the type of energy used in heating (such as electricity, wood, flame, or solar energy), distance of heating, and design of the food device, which can further help foster the production of PAHs in food products (33). 

## Conclusion

In this study, a validated method based on QuEChERS sample preparation was applied for analysis of 24 PAHs in various traditional and semi-industrial Taftoon bread samples in Iran. For overcoming matrix effect, spiked calibration curves were used. According to the EU criteria, correlation coefficient, recovery percentage, and precision were acceptable for determination of PAHs in Taftoon bread. Analysis of Taftoon samples showed that three compounds; NPH, PHE, and ANT were found in 37 (35.2%) Taftoon samples. All of the obtained results were higher than the legal permissible limits (1.0 ng/g) proposed by the European Union for processed cereal-based foods. Our results showed that the detected PAHs in bread samples could be originated from gas oven.
